# Tranexamic Acid Use in Total Hip Arthroplasty for Avascular Necrosis: A Single-Center Experience

**DOI:** 10.3390/jcm14103399

**Published:** 2025-05-13

**Authors:** Wojciech Konarski, Michał Derczyński, Kamil Poboży, Julia Domańska-Poboża, Tomasz Poboży

**Affiliations:** 1 Medical Rehabilitation Center, Sobieskiego 47D, 05-120 Legionowo, Poland; 2 Independent Researcher, 05-123 Olszewnica Stara, Poland; michalderczynski@gmail.com (M.D.); tomasz.pobozy@onet.pl (T.P.); 3 Department of Neurosurgery, Brodnowski Masovian Hospital, 03-242 Warsaw, Poland; pobozykamil@gmail.com; 4 Department of Rheumatology, National Institute of Geriatrics, Rheumatology and Rehabilitation, 02-637 Warsaw, Poland; julia-domanska03@wp.pl

**Keywords:** tranexamic acid, total hip arthroplasty, avascular necrosis, perioperative blood loss, blood transfusion, orthopedic surgery

## Abstract

**Background:** Avascular necrosis (AVN) of the femoral head is a major indication for total hip arthroplasty (THA), often associated with significant blood loss and high transfusion rates. Tranexamic acid (TXA) has been shown to reduce perioperative bleeding, but evidence in AVN-specific populations remains limited. **Methods:** This retrospective, single-center study analyzed 115 patients undergoing primary THA due to AVN between 2016 and 2023. Patients who received TXA were compared with those who did not. Baseline and perioperative data including hemoglobin (HGB), erythrocyte count (RBC), transfusion rates, and PRBC unit use were collected. **Results:** Baseline characteristics were comparable between groups. TXA significantly reduced transfusion incidence (7.9% vs. 36.5%, *p* < 0.0001) and total PRBC unit use (0.1 ± 0.3 vs. 0.8 ± 1.1, *p* < 0.0001). The mean HGB drop was smaller in the TXA group (2.1 ± 1.2 vs. 3.2 ± 2.0 g/dL, *p* = 0.001), as well as the RBC drop (0.8 ± 0.4 vs. 1.3 ± 1.4 million/μL, *p* = 0.02). **Conclusions:** TXA effectively reduces blood loss and transfusion needs in AVN-related THA, supporting its routine perioperative use in this patient population.

## 1. Introduction

Avascular necrosis (AVN) of the femoral head is a form of aseptic osteonecrosis resulting from an impaired blood supply to the proximal femur, leading to osteocyte death. The precise pathogenesis of AVN remains uncertain, with various contributing factors likely playing a role in each individual case. These may include predisposing medical conditions or medications that promote vascular blockage, disruptions in osteocyte metabolic activity, and, potentially, genetics. Mostly, AVN can develop due to ischemic changes, either following trauma or without an obvious injury. The most frequent causes include prolonged corticosteroid use, hip fractures, joint dislocations, and excessive alcohol consumption. AVN of the femoral head most commonly occurs in individuals aged 20 to 40 who are physically active. In the United States, an estimated 10,000 to 20,000 new cases of AVN are identified annually. In the United Kingdom, it is the third most common indication for total hip arthroplasty (THA) in patients younger than 50 years [1,2,3,4,5,6,7]. In the United States, more than 450,000 THA procedures are performed each year [8]. While AVN remains a significant indication for THA, the overall projected increase in THA volume—estimated to rise by approximately 171% by 2030—is primarily driven by multiple factors, including the aging population, rising prevalence of osteoarthritis, and improved surgical access [9].

Management of AVN begins with conservative approaches, such as activity modification and weight-bearing restrictions, primarily recommended in early-stage, non-traumatic cases. Pharmacological options, including bisphosphonates, statins, vasodilators, and anticoagulants, aim to delay disease progression but lack robust clinical guidelines due to limited evidence. Non-invasive modalities like extracorporeal shockwave therapy and hyperbaric oxygen therapy have shown potential benefits in early stages. When conservative measures fail, joint-preserving surgical procedures such as core decompression, nonvascularized or vascularized bone grafting, osteotomies, and stem cell-based therapies may be considered, particularly in younger patients. THA remains the definitive treatment for advanced-stage AVN with femoral head collapse and functional impairment, offering reliable pain relief and restoration of joint function [1,10,11,12,13].

THA carries a high risk of significant blood loss due to surgical bleeding, additional hidden blood loss into the tissues, and increased hemolysis. Perioperative anemia is associated with higher mortality rates. Blood transfusion is required in up to one-third of cases, leading to various complications, including allergic reactions, infection risk, transfusion-related acute lung injury (TRALI), sepsis, kidney injury, prolonged hospital stay, and increased procedural costs [14,15,16,17]. Research on blood management after THA has explored different strategies to minimize blood loss and decrease the need for allogeneic blood transfusions.

Tranexamic acid (TXA) is a synthetic lysine analog that inhibits fibrinolysis by competitively blocking plasminogen activation, preventing its conversion to plasmin, the enzyme responsible for fibrin clot degradation. This stabilization of blood clots reduces intraoperative and postoperative bleeding, making TXA an effective agent for blood conservation in surgical procedures [18,19,20,21]. TXA has been shown to significantly reduce perioperative blood loss and the need for blood transfusions in THA. Excessive blood loss during surgery can lead to perioperative anemia, increased transfusion rates, and longer hospital stays, all of which contribute to higher healthcare costs and greater postoperative risks [22,23]. Research has indicated that, despite a slight increase in pharmacy costs, TXA use leads to a lower total hospitalization cost [24,25,26]. Additionally, TXA has been found to eliminate the need for homologous blood transfusions and significantly reduce the volume of autologous transfusions. This decrease in blood loss enables a more efficient use of intraoperative blood salvage systems and helps minimize the financial burden associated with blood conservation. Furthermore, TXA therapy has been reported as safe, with no significant increase in thromboembolic complications, making it a valuable and cost-effective tool in perioperative blood management protocols for THA [27,28,29].

The aim of this study was to evaluate the efficacy and safety of TXA in reducing blood loss and the need for blood transfusion in patients undergoing primary THA due to AVN. The primary outcome was the proportion of patients requiring transfusion. Secondary outcomes included perioperative changes in HGB and RBC levels. This single-center, retrospective study was designed to assess these effects in a homogenous AVN patient population.

## 2. Materials and Methods

### 2.1. Study Design

This study is a single-center, retrospective observational analysis conducted in the Department of Trauma and Orthopedic Surgery at the Voivodeship Specialist Hospital in Ciechanów, Poland. Eligible participants were identified based on medical records and surgical databases. This study included all consecutive adult patients who underwent primary THA due to AVN in stage III or IV, as classified by the Ficat system, between 2016 and 2023. Patients were included regardless of whether they had administered TXA, allowing comparison between the TXA and non-TXA groups. The non-TXA group included individuals who had potential contraindications such as a history of thromboembolic events, known hypersensitivity to TXA, or other clinical concerns. In some cases, patients declined consent for its use. This study was conducted following ethical guidelines and local institutional review board regulations. All patients provided written consent for the procedure.

### 2.2. Data Collection

The collected data included preoperative and postoperative hemoglobin levels, erythrocyte counts, patient age, sex, body mass index (BMI), required number of transfusions, and preoperative coagulation parameters, including international normalized ratio (INR) and activated partial thromboplastin time (APTT).

### 2.3. Surgical and Pharmacological Protocol

All procedures were performed via a posterior-lateral approach following standard aseptic surgical techniques. Patients received TXA intravenously at a total dose of 2 g, with 1 g administered immediately before surgery and an additional 1 g during skin closure at the end of the procedure. Thromboprophylaxis was standardized, with enoxaparin (0.4 mL subcutaneously) given at least 12 h before surgery to minimize the risk of thromboembolic events. Thromboprophylaxis after surgery included enoxaparin administered 12 h postoperatively and then every 24 h until hospital discharge. Upon discharge, further anticoagulant management was determined individually by the attending physician.

### 2.4. Blood Management and Transfusion Protocol

Postoperative hemoglobin (HBG) levels were closely monitored. Blood transfusion was indicated if HBG levels dropped below 8.9 g/dL or if the total reduction exceeded 30% of baseline HBG, accompanied by clinical symptoms such as pallor, weakness, or drowsiness. Postoperative laboratory assessments were conducted in accordance with standard procedures at our center. Routine blood tests were typically performed 24 h after surgery. However, in cases where the patient’s clinical condition appeared compromised—manifested by signs such as pallor, weakness, or visible symptoms of anemia—blood testing was permitted as early as 12 h postoperatively to ensure timely evaluation and intervention.

### 2.5. Statistical Analysis

Descriptive statistics were used to summarize baseline and perioperative characteristics. Continuous variables were expressed as mean ± standard deviation (SD) and compared between groups using Welch’s *t*-test, which does not assume equal variances. Categorical variables, including sex and transfusion status, were compared using the chi-square test.

For perioperative outcomes, the differences in hemoglobin and erythrocyte levels before and after surgery were calculated, and mean changes were compared using Welch’s *t*-test. Due to the non-normal distribution of transfusion-related data (number of patients receiving transfusion and number of PRBC units), comparisons between groups were performed using the non-parametric Mann–Whitney U test.

A post hoc power analysis was conducted based on the primary outcome (proportion of patients requiring transfusion). The analysis demonstrated that this study had a statistical power of 97.3% to detect the observed difference between groups (α = 0.05, two-sided test), suggesting an adequate sample size for this outcome.

All statistical analyses were performed using Statistica v.13 software (StatSoft, Kraków, Poland). A *p*-value of less than 0.05 was considered statistically significant.

## 3. Results

### 3.1. Baseline Characteristics

The baseline characteristics of the patients are summarized in Table 1. Both groups were comparable in terms of demographic and laboratory parameters prior to surgery. A total of 115 patients undergoing primary THA were included in this study; 63 received TXA during the perioperative period, while 52 did not. All the procedures were performed without complications, with standard operative time and no major intraoperative blood loss. There were two cases of autologous transfusion in total—one in each group.

The mean age was similar between the TXA group (63.3 ± 11.1 years) and the non-TXA group (63.1 ± 10.0 years; *p* = 0.90). No significant differences were observed in BMI (28.2 ± 3.9 vs. 29.3 ± 4.1 kg/m^2^; *p* = 0.18), baseline INR (1.04 ± 0.07 vs. 1.03 ± 0.06; *p* = 0.61), or baseline APTT values (28.9 ± 4.1 vs. 27.7 ± 3.2 s; *p* = 0.09).

The distribution of sex was also balanced between groups, with 29 females (46.0%) and 34 males (54.0%) in the TXA group and 23 females (44.2%) and 29 males (55.8%) in the non-TXA group. There was no statistically significant difference in the sex distribution between the two groups (*p* = 0.86, chi-square test).

### 3.2. Effects of Tranexamic Acid on Perioperative Blood Parameters

**Figure 1:**
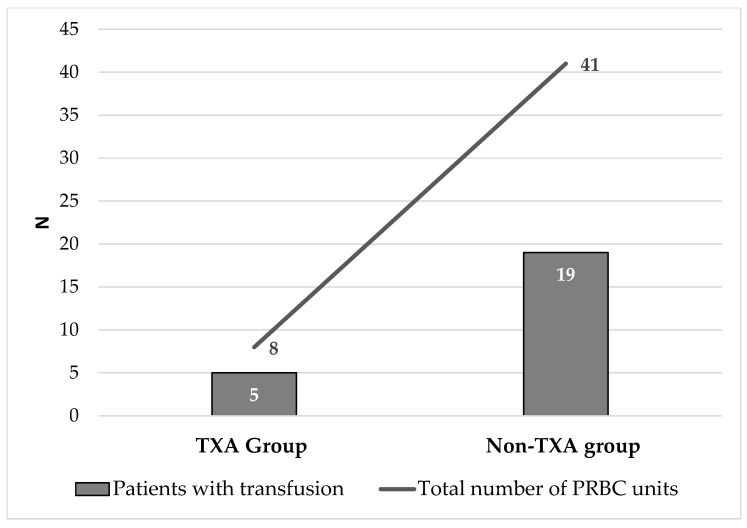
The effect of tranexamic (TXA) acid on the need for blood transfusion and the number of packed red blood bell (PRBC) units transfused.

The perioperative outcomes are summarized in Table 2. Only 5 (7.9%) of the patients in the TXA group required transfusion, compared to 19 (36.5%) in the non-TXA group (*p* < 0.0001). Similarly, the total number of PRBC units transfused was significantly lower with TXA (0.1 ± 0.3 vs. 0.8 ± 1.1, *p* < 0.0001) (Figure 1).

The mean hemoglobin drop was significantly smaller in the TXA group (2.1 ± 1.2 g/dL) than in the control group (3.2 ± 2.0 g/dL, *p* = 0.001). A similar pattern was observed for erythrocyte count, with a mean decrease of 0.8 ± 0.4 million/μL in the TXA group versus 1.3 ± 1.4 million/μL in controls (*p* = 0.02).

**Figure 2:**
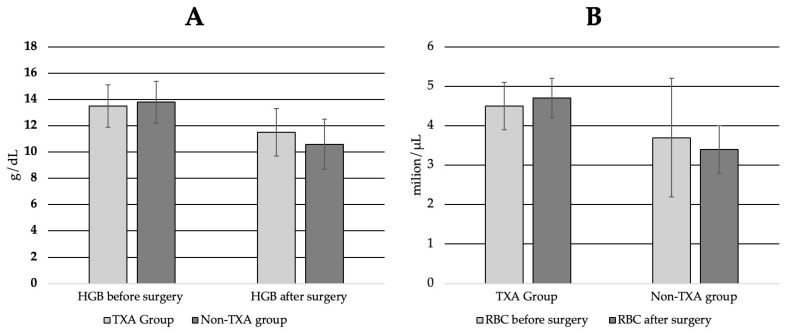
The effect of tranexamic acid (TXA) on (**A**) hemoglobin levels and (**B**) red blood cell (RBC) count. The error bars represent the standard deviation.

Although preoperative hemoglobin and red blood cell levels were comparable between groups (HGB: 13.5 ± 1.6 vs. 13.8 ± 1.8 g/dL; RBC: 4.5 ± 0.6 vs. 4.7 ± 1.5 million/μL), postoperative values were significantly higher in the TXA group (HGB: 11.5 ± 1.6 vs. 10.6 ± 1.9 g/dL, *p* = 0.01; RBC: 3.7 ± 0.5 vs. 3.4 ± 0.6 million/μL, *p* = 0.007) (Figure 2).

In our study, no adverse reactions thromboembolic complications were observed in either the TXA or non-TXA group.

## 4. Discussion

This single-center retrospective study demonstrated that the use of TXA in patients undergoing THA for AVN was associated with significantly reduced perioperative blood loss and transfusion requirements. Patients who received TXA had a lower incidence of transfusion, required fewer PRBC units, and showed smaller decreases in hemoglobin and erythrocyte levels compared to those who did not receive TXA. Importantly, baseline characteristics between the TXA and non-TXA groups were comparable, strengthening the reliability of the observed outcomes. These findings support the efficacy and safety of TXA as part of a perioperative blood management strategy in patients undergoing THA due to AVN.

Our findings are consistent with current literature supporting the use of TXA in THA. In a comprehensive network meta-analysis by Fillingham et al. [22], strong evidence was demonstrated for the efficacy of TXA in reducing both blood loss and the need for transfusion following primary THA. The study included 34 trials and showed that TXA in different formulations, topical, intravenous, and oral, were effective when compared to placebo. However, no particular route, dose, or timing strategy was found to be clearly superior. These results have supported major clinical practice guidelines from leading orthopedic and anesthesiology societies. Similar to those findings, our study showed that TXA significantly reduced transfusion rates and perioperative decreases in hemoglobin and erythrocyte levels in patients with AVN. In an umbrella review by Ghorbani et al. [30], the efficacy and safety of TXA THA were comprehensively evaluated based on 23 meta-analyses covering data from over 35,000 patients. The findings demonstrated that TXA significantly reduces perioperative blood loss (by 151–370 mL), postoperative hemoglobin drop (by 0.5–1.1 g/dL), and transfusion requirements (by 19–26%) compared to control groups. Notably, TXA administration was not associated with an increased risk of venous thromboembolism or wound complications. Furthermore, no substantial differences in outcomes were found across different dosages, modes of administration, or when TXA was used in combination with other antifibrinolytic agents. These results reinforce the favorable risk–benefit profile of TXA in THA and support its routine use in clinical practice.

Additional insights into the safety profile of TXA in THA have been provided by a recent large-scale retrospective cohort study by Thapaliya et al. [31] utilizing the TriNetX Research network, which included adult patients undergoing THA between 2003 and 2024. The analysis compared outcomes between patients receiving TXA within 24 h prior to surgery and those who did not. At both 30 and 90 days postoperatively, TXA was associated with a significantly reduced risk of transfusion, deep vein thrombosis, and periprosthetic joint infection. However, the study also reported higher rates of periprosthetic fractures (RR: 1.2; 95% CI: 1.09–1.36), acute postoperative anemia, and both superficial and deep surgical site infections in the TXA cohort. These findings suggest that, while TXA remains a highly effective agent for minimizing blood loss and thromboembolic risk, its administration may be associated with specific postoperative complications that need further investigation. Future research should aim to clarify the clinical significance of these findings and identify patient subgroups that may be at higher risk for such adverse events.

Reducing the need for allogeneic blood transfusion offers several well-established benefits. Clinically, transfusion avoidance reduces the risk of transfusion-related complications such as febrile non-hemolytic reactions, hemolysis, allergic responses, TRALI, immunomodulation, and transmission of infections such as HIV or hepatitis viruses [15,16,17]. Some studies have also reported a correlation between perioperative transfusion and increased rates of postoperative infections and delayed recovery. Limiting transfusion demand reduces the burden on blood bank logistics and improves work efficiency in perioperative care [14].

In our study, no adverse events or thromboembolic complications were observed in the studied patients. This supports the safety profile of TXA, which has been consistently demonstrated in the literature, particularly regarding its low risk of deep vein thrombosis or pulmonary embolism when used in the context of orthopedic surgery [32,33].

In our study, all the patients who received TXA were administered the drug intravenously as part of the perioperative protocol. However, recent evidence suggests that topical application of TXA may offer similar efficacy. A meta-analysis by Artykbay et al. [34], which included nine randomized controlled trials with 1024 patients undergoing hip fracture surgery, demonstrated that topical TXA significantly reduced hemoglobin loss (mean difference [MD], 1.00 g/dL *p* = 0.03) and the number of transfused blood units (relative risk, 0.64; *p* = 0.001) compared to placebo. Importantly, the analysis found no significant differences between topical and intravenous TXA in terms of transfusion rates, total blood loss, deep vein thrombosis incidence, or surgical duration. In a subgroup of patients undergoing arthroplasty, topical TXA was shown to significantly reduce hemoglobin drop (MD, 1.50 g/dL; 95% CI, 0.32–2.68; *p* = 0.012) and total blood loss (MD, –322.3 mL; 95% CI, –566.6 to –78.0; *p* = 0.010) when compared to placebo. These findings suggest that both intravenous and topical TXA may be effective strategies for blood conservation in hip arthroplasty, and further comparative studies are warranted to define the optimal route of administration.

Beyond its well-established role in reducing perioperative blood loss, TXA may also exert anti-inflammatory effects, which could further benefit postoperative recovery. Recent evidence suggests that TXA administration, particularly in multi-dose regimens, can attenuate systemic inflammatory responses after joint arthroplasty. A meta-analysis by Rui et al. [35], including nine randomized controlled trials in patients undergoing hip and knee arthroplasty, demonstrated that multi-dose TXA significantly reduced levels of inflammatory markers such as IL-6 and CRP compared to lower-dose protocols. Additionally, the use of multi-dose TXA was associated with a shorter length of hospital stay, with no significant increase in thromboembolic complications. These findings suggest that the benefits of TXA may extend beyond hemostatic control, potentially contributing to improved postoperative outcomes through modulation of inflammation. However, high-quality, large-scale studies are warranted to confirm and better understand the clinical significance of these effects.

Finally, strategies such as TXA administration, which significantly lower transfusion incidence, contribute not only to improved patient outcomes but also to more cost-effective and resource-conscious perioperative care. Although TXA adds a small pharmacy cost, its use has been consistently associated with overall cost savings in THA. By effectively reducing perioperative blood loss and minimizing the need for allogeneic blood transfusions, TXA contributes to lower expenditures related to transfusion products, laboratory monitoring, and extended hospitalization. In the study by Gillette et al. [25], TXA increases pharmacy cost slightly (e.g., USD 921 vs. USD 781, *p* < 0.0001); its routine use has been shown to reduce overall direct hospital cost (e.g., USD 15,099 with TXA vs. USD 15,978 without, *p* < 0.0002). The retrospective study by Irisson et al. [24], which compared patients undergoing hip and knee arthroplasty before and after the implementation of a standardized TXA protocol, demonstrated a marked reduction in blood transfusion requirements. The use of TXA led to a complete elimination of homologous transfusions, a 34% decrease in total blood loss, and reductions of 38% and 68% in the rate and volume of autologous transfusions, respectively. Despite the added cost of TXA administration, overall blood conservation strategy costs were reduced by 25%, primarily due to decreased reliance on blood salvage systems and transfused blood products. These findings underscore the dual clinical and economic value of TXA and support its routine use in orthopedic surgery as a cost-saving measure that also enhances patient safety. In our study, patients receiving TXA required fewer transfusions (5 vs. 19) and fewer PRBC units (8 vs. 41), supporting its cost-effectiveness.

This study has several limitations. First, its retrospective design and single-center setting may limit the generalizability of the findings. Second, the absence of randomization introduces a potential for selection bias, despite comparable baseline characteristics between groups. To further validate these results, a prospective randomized controlled trial is planned to more rigorously assess the efficacy and safety of TXA in patients undergoing THA for AVN.

Future research should further explore the use of TXA in AVN-specific populations, particularly in comparison with other indications for THA, such as osteoarthritis or femoral neck fractures. Given the vascular etiology of AVN, it should be acknowledged that patients with this condition may respond differently to antifibrinolytic therapy. Stratifying outcomes by AVN stage or etiology (e.g., steroid-induced vs. alcohol-related) may also provide beneficial insights into the effectiveness and safety of TXA. Moreover, the potential anti-inflammatory effects of TXA, which have been suggested in recent meta-analyses, warrant investigation in the AVN setting, where inflammation and bone turnover may influence surgical outcomes. Multicenter, prospective studies with larger sample sizes and standardized TXA protocols would help confirm the findings of our single-center study and provide more robust evidence to guide perioperative management in this specific patient group. Although this study was retrospective in design, post hoc power analysis confirmed sufficient statistical power (97.3%) to detect the difference in transfusion rates between groups. While further multicenter studies are warranted, our findings provide robust preliminary evidence supporting the efficacy of TXA in AVN-related THA.

## 5. Conclusions

The use of TXA in patients undergoing THA for AVN was associated with a significant reduction in perioperative blood loss and transfusion requirements. Patients who received TXA experienced a smaller postoperative drop in HGB and RBC levels, indicating more effective hemostatic control during and after surgery. Additionally, fewer patients in the TXA group required transfusion, and the overall number of PRBC units administered was substantially lower compared to those who did not receive TXA. Importantly, no increase in thromboembolic events or other complications was observed, supporting the favorable safety profile of TXA in this setting. These findings reinforce the role of TXA as an effective and safe component of perioperative blood management strategies in patients undergoing THA for AVN. Routine use of TXA in this population may contribute to better clinical outcomes, reduced reliance on transfusion, and improved overall perioperative care and economy.

## Figures and Tables

**Table 1:** Baseline characteristics.

	TXA Group (N = 63)	Non-TXA Group (N = 52)	*p*-Value
Age (years), mean (SD)	63.3 (11.1)	63.1 (10.0)	0.90
Sex, % Women	46%	44%	0.86
BMI (kg/m^2^), mean (SD)	28.2 (3.9)	29.3 (4.1)	0.18
INR	1.0 (0.1)	1.0 (0.1)	0.61
APTT (s)	28.9 (4.1)	27.7 (3.2)	0.09

APTT—activated partial thromboplastin time; BMI—body-mass index; INR—international normalized ratio; SD—standard deviation; TXA—tranexamic acid.

**Table 2:** Perioperative outcomes in patients with and without tranexamic acid (TXA) administration.

	TXA Group (N = 63)	Non-TXA Group (N = 52)	*p*-Value
Patients with transfusion N (%)	5 (7.9%)	19 (36.5%)	<0.0001
Total number of PRBC units	8	41	<0.0001
HGB before surgery (g/dL) (SD)	13.5 (1.6)	13.8 (1.8)	0.46
HGB after surgery (g/dL) (SD)	11.5 (1.6)	10.6 (1.9)	0.01
Mean decrease in HGB (g/dL) (SD)	2.1 (1.2)	3.2 (2.0)	0.001
RBC before surgery (million/μL) (SD)	4.5 (0.6)	4.7 (1.5)	0.30
RBC after surgery (million/μL) (SD)	3.7 (0.5)	3.4 (0.6)	0.007
Mean decrease in RBC (million/μL) (SD)	0.8 (0.4)	1.3 (1.4)	0.02

HGB—hemoglobin; RBC—red blood cell; SD—standard deviation; TXA—tranexamic acid.

## Data Availability

The data that support the findings of this study are available on request from the corresponding author. The data are not publicly available due to their containing information that could compromise the privacy of research participants.
